# Knowledge, Attitudes, and Practices towards Diabetic Retinopathy among Primary Care Physicians of Saudi Arabia: A Multicenter Cross-Sectional Study

**DOI:** 10.3390/healthcare9121697

**Published:** 2021-12-08

**Authors:** Ashokkumar Thirunavukkarasu, Abdulmohsen Khaled Almulhim, Faisal Ahmed Albalawi, Ziyad Muharib Alruwaili, Ola Ali Almajed, Sultan Hamoud Alruwaili, Malek Mohammed Almugharriq, Abdulaziz Saud Alruwaili, Malak Khalid Alkuwaykibi

**Affiliations:** 1 Department of Community and Family Medicine, College of Medicine, Jouf University, Sakaka 72388, Saudi Arabia; 2 Department of Ophthalmology, College of Medicine, Jouf University, Sakaka 72388, Saudi Arabia; Akalmulhim@ju.edu.sa; 3 College of Medicine, Jouf University, Sakaka 72388, Saudi Arabia; faisalrmzr@hotmail.com (F.A.A.); ziad623@gmail.com (Z.M.A.); olalmajed2@gmail.com (O.A.A.); mkln2012@hotmail.com (S.H.A.); maalik2999@gmail.com (M.M.A.); alnosairy.9@gmail.com (A.S.A.); 4 Department of Ophthalmology, King Abdulaziz Specialist Hospital, Sakaka 72345, Saudi Arabia; dr.malak1695@gmail.com

**Keywords:** diabetic retinopathy, knowledge, attitude, practices, primary care

## Abstract

Primary care physicians play a vital role in preventing the progression of diabetic retinopathy (DR) from the initial stages to the late stages. This questionnaire-based analytical cross-sectional study was carried out to assess the knowledge, attitude, practices, and their correlation among 274 randomly selected primary care physicians in Saudi Arabia. Among the studied population, high knowledge, attitudes, and practice scores were observed in 21.5%, 15%, and 29.2% of the physicians, respectively. The mean knowledge score was significantly higher among the age group of less than 30 years (*p* = 0.031) and the female gender (*p* = 0.012). The attitude scores were significantly higher among the Saudi physicians (*p* = 0.027) and those with PHC work experience of less than five years (*p* < 0.001). Regarding the practices, a significant association was found among the age group of less than 30 years (*p* = 0.019) and Saudi physicians (*p* = 0.003). There was a significant positive correlation between knowledge (correlation coefficient (r) = 0.739, *p* < 0.001) and attitude (r = 0.479, *p* = 0.007) with the practice scores. It is recommended that targeted continuous medical education, workshops, and seminars are conducted on the prevention and care of DR among primary care physicians. Furthermore, an exploratory multicenter study that involves primary care physicians belonging to all ministries and private sectors is warranted.

## 1. Introduction

The International Diabetes Federation (IDF) reported that the prevalence of diabetes mellitus (DM) in the adult population in the Kingdom of Saudi Arabia (KSA) is 18.3%, and the total cases of DM are estimated to be around 4.3 million among the adult population [1]. DM can lead to several complications and diabetic retinopathy (DR) is one of the most common complications of DM [2,3]. DR is considered to be the most common and serious microvascular complication that leads to a visual impairment and blindness among the working age group worldwide [4,5,6]. There are numerous reasons for blindness and a visual impairment among DR patients such as diabetic maculopathy, a vitreous hemorrhage, retinal detachment, and neurovascular glaucoma [2].

Globally, the prevalence of DR ranges from 18.9% to 40.3% among diabetes patients [7,8,9]. A study performed in the Taif region by Al Ghamdi et al. found that the prevalence of DR among the patients aged 50 years and above was 36.8% [10]. Another study performed by Hajar et al. in the southern region of the KSA reported that 28.6% of DM patients were affected by DR [11]. Although all patients with diabetes have a risk of developing DR, there are several methods by which DR can be controlled such as the adequate management of glycemic control, hypertension, and serum cholesterol control [3,12].

Health care is delivered in the KSA at four levels, starting from primary health centers and going up to referral hospitals. Primary health care is delivered in the KSA through a chain of primary health centers and this is the first level of contact for the public for all their health-related needs [13]. The primary care physicians are essential components of DM management and the follow-up of the patients as they are the first line and in direct contact with the local community. They cater for the majority of health needs of people including DM and its associated complications [14].

In the initial stages of DR, patients are usually asymptomatic and rarely notice their vision impairment. Hence, they may not seek clinical attention from physicians, primarily those not specialized in ophthalmology [8,15]. The prevention of the progression of DR from the initial stages primarily involves coordinated efforts of patients and physicians who manage DM and DR [12]. In the KSA, primary care physicians play a significant role in diagnosing, preventing, managing, and following these patients [13,14]. A survey conducted by Alsaqah et al. in 2020 among primary care physicians reported that the knowledge of primary care physicians of DR was suboptimal and they recommended undertaking future exploratory research to find the associated factors for poor knowledge among primary care physicians [16]. Another study by Abdulsalam et al. reported that only 36.7% of participants practiced a regular ophthalmic examination among diabetic patients. They did not find a significant correlation between the knowledge, attitude, and practices among physicians [17]. A study conducted in the Al-Hasa region of the KSA in 2020 reported that mean knowledge scores were not significantly associated with sex, age group, and the year of medical practice [18]. However, the above studies were surveyed in a single region and/or centers with low sample sizes and non-probability convenience sampling techniques. Furthermore, none of the KSA studies have attempted to find a correlation between the knowledge, attitude, and practice towards DR among primary care physicians.

The assessment of knowledge, attitude, and practices towards DR among patients and primary care physicians is essential for coordinated efforts to prevent the progression of DR from the initial stages to the late stages. In the KSA, several studies have been performed among the patients to assess their awareness and attitude towards DR [19,20]. However, limited data/studies are available among primary care physicians that have explored the associated factors of knowledge, attitude, and practice scores towards DR. Hence, this study was planned to keep in mind the vision of having regional data that can be used to plan and implement healthcare services related to DR. The present study aimed to assess the knowledge, attitude, and practices towards DR among primary care physicians working in different primary health centers, to identify the factors that influence knowledge, attitude, and practices as well as to determine the correlation between knowledge, attitude, and practices towards DR.

## 2. Materials and Methods

### 2.1. Study Design, Setting, and Sample Size Estimation

This analytical cross-sectional study was carried out from June 2021 to October 2021. In the KSA, health care to the public is delivered through four levels: primary health centers, general hospitals, specialty hospitals, and medical cities (apex hospitals). There are thirteen administrative regions/provinces in the KSA. The present study was performed among the primary care physicians working at PHCs in four regions of the KSA (Aljouf, Hail, the Northern Border, and Tabuk). According to the Ministry of Health (MOH), KSA, there were 942 primary care physicians registered in these four regions.

The sample size was calculated based on the formula z^2^ pq/e^2^. In this formula, p is the expected prevalence, q is 1 − p, and e is the margin of error at 5%. Whilst calculating the sample size, the factors considered were the expected proportion (p) as 50%, a study power of 80%, and a confidence interval at 95%. As previous studies have depicted a wide range of prevalence in this subject, we took p as 50% to obtain the maximum sample size. Considering the above factors with a finite population of 942, the estimated sample size was 274.

### 2.2. Sampling Method

The present study applied a probability systematic random sampling method to select the required number of study participants. In this method, all primary care physicians were ordered according to their number assigned by the research team and every third physician was invited to participate in the study. In the event of a non-response from the selected physician, the next participant on the list was asked to participate.

### 2.3. Data Collection Procedure

The research team initiated the data collection process after ethical clearance and other necessary approvals. The data collectors of this research visited the selected physicians at their health centers. After briefing about the study and obtaining informed consent, the participants were requested to fill in the online data collection sheet on the electronic devices of the data collectors (mobile, tablet, and laptop). The research team followed the standard COVID-19 prevention protocol administered by the MOH, KSA, during the data collection process. No other member of the research team except the principal investigator had authorization to access the Google form for the present study. The present study only included MOH-registered physicians. Primary care physicians working in other ministries, physicians on vacation, physicians unwilling to participate, and physicians in the private sector were excluded from this study.

### 2.4. Data Collection Tool (Survey Questionnaire)

A Google form was created with a standard and validated questionnaire adapted from open-source pieces of literature [16]. We conducted a pilot study among 30 physicians using the adapted questionnaire. The pilot study result revealed the Cronbach values of knowledge, attitude, and practice were 0.76, 0.81, and 0.79, respectively, which displayed a good internal consistency of the format used. Hence, we proceeded with the present format of the questionnaire to collect data consisting of four parts. Part 1 contained the socio-demographic characteristics such as age, gender, place of work, and year of experience. Part 2 contained twelve questions related to the knowledge of the physicians towards DR, assessing the prevalence of visual impairments due to DR in the KSA, risk factors, parts of the eye to examine for DR, screening methods, and available modalities to treat DR. Part 3 included nine questions related to the attitude of physicians towards DR, asking about the role of primary care physicians in preventing the progression of DR, periodic examination frequency, and why ophthalmological examinations should be done for DM patients and so on. Part 4 consisted of eleven questions related to the practices of physicians towards DR on performing the ophthalmoscopic examination, regular health education for the patients, the ability to assess the retinal conditions, observing comorbid conditions, follow-up, and the referral of patients in need of further assistance. A 5-point Likert scale with responses from strongly agree to strongly disagree was recorded for the knowledge, attitude, and practice sections. The scores were calculated as one for strongly disagree to five for strongly agree. Therefore, the total knowledge scores ranged from 12 to 60, the total attitude score from 9 to 45, and the total practice score from 11 to 55. The obtained total scores in each section were categorized into low (<60% of the total score), medium (60 to 80% of the total score), and high (>80% of the total score).

### 2.5. Statistical Analysis

The collected Microsoft Excel data from the Google form was exported to Statistical Packages for Social Sciences (SPSS), version 21, and coded as per the coding sheet of this survey. The categorical variables were presented as the frequency and proportion (*n*; %) and the continuous data were presented as a mean and standard deviation (SD). Initially, we performed a Kurtosis and Skewness analysis followed by the Kolmogorov-Smirnov (K-S) test to assess the normality assumption of the collected data. The K-S test values were 0.093, 0.111, and 0.084 and the asymptotic significant values (two-tailed) were 0.158, 0.194, and 0.182, respectively, for the knowledge, attitude, and practices scores. We then performed a one-way analysis of variance (ANOVA) and an independent *t*-test to find the difference between the knowledge, attitude, and barrier scores with the socio-demographic characteristics of the study population. The research team also executed a Pearson’s correlation analysis to find the relationship between the knowledge, attitude, and practices of the primary care physicians towards DR. Furthermore, we performed a multiple linear regression analysis to identify the correlation between the knowledge, attitude, and practices scores after adjustment with other covariables of the present study. A *p*-value less than 0.05 identified through two-tailed tests was considered to be a statistically significant value.

## 3. Results

Table 1 presents the socio-demographic characteristics of the study participants. Of the 274 participants, more than half (54.4%) were male, non-Saudi primary care physicians (52.6%); 42.3% had completed only a bachelor (MBBS) degree and the majority had worked more than 5 years at a PHC (41.2%).

Of the studied population, only a low proportion of the participants had high knowledge (21.5%), attitude (15%), and practice (29.2%) scores. The majority of them had medium/low scores in all three variables (Table 2).

Table 3 shows the relationship between the knowledge, attitude, and practice scores with the socio-demographic characteristics. Of the 274 primary care physicians, the mean knowledge score was significantly higher among the age group of less than 30 years (*p* = 0.031), the female gender (*p* = 0.012), Saudi physicians (*p* < 0.001), and PHC work experience of less than 5 years (*p* = 0.014). The mean attitude scores were significantly higher among the age group of less than 30 years (*p* = 0.001), Saudi physicians (*p* = 0.027), and PHC work experience of less than 5 years (*p* < 0.001). Regarding the practices, a significant association was found between the age group of less than 30 years (*p* = 0.019) and Saudi physicians (*p* = 0.003).

The correlation between the knowledge, attitude, and practice was evaluated by using a Pearson’s correlation analysis. Table 4 demonstrates that there was a significant correlation between both knowledge (r = 0.739, *p* < 0.001) and attitude (r = 0.479, *p* = 0.007) with the practice scores.

After adjusting for gender, age group, current working position, nationality, and PHC work experience duration, there was a significant association between both knowledge (β = 0.658, *p* = 0.008) and attitude (β = 0.539, *p* < 0.038) with the practice scores (Table 5).

## 4. Discussion

World Diabetes Day is held every year on 14 November by the United Nations (UN), WHO, and IDF with the main focus on raising awareness of diabetes and its complications. The UN declared the theme for World Diabetes Day 2021–2023 as “access to diabetes care”, focusing on the necessity of continuous support and care to manage the diabetes status and avoid complications such as DR [21]. Primary care physicians play an important role in achieving the above goal of the UN as they provide care to approximately 90% of diabetes patients [22]. The present study aimed to assess the knowledge, attitude, and practice of primary care physicians and to assess the relationship between the knowledge and attitude with the practices towards DR. 

The present study revealed a suboptimal knowledge among primary care physicians about DR. Similar to the results of the present study, a study by Nyonsavye in Kenya also reported that only a low proportion of general practitioners and primary care physicians had a high knowledge of DR [23]. Similarly, Khandekar et al. reported that 40% of physicians in Oman were graded poorly in a few items of the knowledge category related to DR [24]. Interestingly, a survey conducted in Nigeria in 2018 reported a high knowledge among physicians towards DR. This difference could be due to settings and differences in the inclusion of participants [17]. The latter study included all non-ophthalmology physicians including family medicine, internal medicine, and other doctors handling diabetes patients whereas the present study included only primary care physicians. The present study revealed that the knowledge score towards DR was significantly higher among the younger age group and Saudi primary care physicians. Interestingly, a study performed by Alhejji et al. in 2020 in the Al Hasa region of the KSA found that the knowledge score was not significantly associated with age and nationality [18]. These differences could be due to study settings and the proportion of each nationality. In our study, more than half of the study participants were non-Saudi physicians whereas the latter included only 24.1% of non-Saudi physicians. This study depicted a significant association between the duration of work experience at a PHC and the knowledge score. Similar to our study, Al Rashid et al., on the assessment of the knowledge, attitude and practices towards DR among primary care physicians of the Riyadh region, KSA, also revealed a significant association between the knowledge scores and work experience [25]. In contrast to the current study, Wiggins et al. reported no differences in the knowledge score according to the years of practice at a PHC [26]. The other possible reasons for the significant association of the younger age group, males, and physicians with less than five years of experience with the knowledge, attitude, and practice towards DR could be cultural settings and the preferences of physicians for selecting postgraduation programs. In the KSA, physicians can apply for a postgraduate residency training program every year through a structured examination conducted by the Saudi Commission for Health Specialties. The majority of the accepted applicants are male, Saudi, and young physicians. The above statement is supported by another study conducted by Khan et al. in the KSA. Their study showed that the knowledge, attitude, and practice scores towards type 2 diabetes mellitus were significantly higher among the males and the physicians with less experience (1–5 years) [27]. 

The present study further attempted to find the association between the socio-demographic characteristics with the attitude and practice scores. This study found that both the attitude and practice scores towards DR were significantly higher among the younger age group and Saudi nationals. Furthermore, the present study did not reveal an association between male and female genders with the attitude and practice scores. Contrary to the present study findings, a study performed in the Riyadh region of the KSA found no association between age and nationality [28]. Interestingly, a study undertaken by Alharbi et al. found that the practice scores were significantly associated with the gender of their study participants [29]. However, these contrasting results could be due to the tools they used and the inclusion of participants. The latter study included all non-ophthalmic healthcare providers from the private sector whereas our study included primary care physicians working in MOH, KSA. 

This study found that only a low proportion of the participants had high knowledge, attitude, and practice scores. Similar to our study, Alrashid et al. in the Riyadh region of the KSA also found that a low proportion of participants had high scores in the knowledge category [25]. In addition, they also found a negative attitude and poor practice of non-ophthalmic healthcare providers towards the eye care of DM patients. It is noteworthy to mention here that healthcare delivery is a knowledge-driven process and a lack of adequate knowledge may lead to inefficient healthcare delivery [30]. This low level of knowledge could drive patients with DM to come to DR care at a later stage where only limited treatment options are available to save their vision. On the other hand, another study conducted in the KSA by Alharbi et al. in 2020 found a higher proportion of participants had good knowledge (57.5%), attitude (89.5%), and practice (79.1%). In addition, they revealed that no socio-demographic factors were significantly associated with the good knowledge, attitude, and practice [29]. A study conducted in China stated that the knowledge of physicians towards eye complications (DR, glaucoma) of DM was generally good. However, their study design was a focused group discussion and performed among 22 physicians only [31].

The current study found a positive correlation between the knowledge and attitude with the practice, In Nigeria, a survey conducted by Abdulsalam et al. reported no significant correlation between the knowledge, attitude, and practice scores [17]. This striking difference could be possibly due to the study setting; the latter was executed in three different tertiary care hospitals. From an extensive review of the literature, the research team could not find any study that attempted to find the correlation between knowledge, attitude, and practices towards DR among primary care physicians in the KSA. Hence, we could not make a comparison with the existing literature in this context with the local settings.

The strength of the present study was that it was conducted among 274 primary care physicians from four different provinces of the KSA. To date, this study has the highest number of primary care physicians surveyed on this subject in the KSA. The research team collected the data using a structured and validated questionnaire. Additionally, we used a probability systematic sampling method to select the required number of participants. Hence, the study has several strengths of systematic sampling methods. Although the research team performed this study with a standard methodology, certain constraints need to be considered whilst judging the results of this study. Firstly, the present study included only primary care physicians working at MOH primary health centers of four regions. Hence, the findings of this study cannot be generalized to all regions of the KSA or the Middle East. Secondly, this cross-sectional study assessed only the association between the variables and not the causation. Finally, this survey was based on self-reported responses from primary care physicians. Hence, the possibility of recall bias and social desirability bias cannot be ignored.

## 5. Conclusions

The present study showed that less than one-third of primary care physicians had a high level of knowledge, attitude, and practice with respect to diabetic retinopathy. In this study, we found several associated factors for high knowledge, attitude, and practices. Furthermore, a positive correlation of the knowledge and attitude with the practice score indicated that an increased knowledge and attitude could help with excellent and efficient practices for DR care at an early stage. Hence, it is recommended that focused continuous medical education (CME), workshops, and seminars are conducted on the prevention and care of DR among primary care physicians. These training programs may also be specifically targeted towards the low and medium knowledge category physicians. Furthermore, an exploratory multicenter study that involves all non-ophthalmic physicians of both the government and private sectors is warranted.

## Figures and Tables

**Table 1:** Socio-demographic characteristics of the primary care physicians. The data presented here are frequency and percentage (*n*, %), (*n* = 274).

Variables	Frequency (*n*)	%
Age group (in years)		
<30	84	30.7
30–40	97	35.4
>40	93	33.9
Gender		
Male	149	54.4
Female	125	45.6
Qualification		
Bachelor (MBBS)	116	42.3
Masters	34	12.4
Fellowship	70	25.5
Saudi board	54	19.7
Nationality		
Saudi	130	47.4
Non-Saudi	144	52.6
PHC work experience		
<5 years	113	41.2
5 to 10 years	84	30.7
>10 years	77	28.1
Current position		
Resident	164	59.9
Specialist	77	28.1
Consultant	33	12.0

**Table 2:** Knowledge, attitude, and practice scores of participants regarding diabetic retinopathy (DR) (*n* = 274).

Variables	Frequency (*n*)	%
Knowledge		
High	59	21.5
Medium/low	215	78.5
Attitude		
High	41	15.0
Medium/low	233	85.0
Practice		
High	80	29.2
Medium/low	194	70.8

**Table 3:** Relationship between the knowledge, attitude, and practice scores of diabetic retinopathy (DR) and the socio-demographic characteristics of the study participants.

Variables	Knowledge			Attitude			Practice		
	Mean ± SD	*p*-Value	Effect Size	Mean ± SD	*p*-Value	Effect Size	Mean ± SD	*p*-Value	Effect Size
Age group (years) *									
<30	47.75 ± 5.48	0.031		32.20 ± 6.81	0.001		44.40 ± 4.92	0.019	
30–40	45.89 ± 5.95		0.88	30.80 ± 5.24		0.56	42.78 ± 5.69		0.37
>40	42.76 ± 5.10			29.01 ± 4.28			42.43 ± 4.94		
Gender **									
Male	46.38 ± 6.18	0.012	0.3	30.84 ± 6.08	0.561	0.07	43.72 ± 5.51	0.086	0.19
Female	44.58 ± 5.49			30.44 ± 5.22			42.69 ± 4.37		
Qualification *									
Bachelor (MBBS)	45.11 ± 4.44	0.072	0.14	30.11 ± 4.95	0.357	0.21	42.29 ± 4.05	0.021	0.66
Masters	45.15 ± 6.95			28.70 ± 3.44			42.21 ± 4.24		
Fellowship	43.51 ± 5.10			31.91 ± 5.62			45.62 ± 6.11		
Saudi board	46.67 ± 5.71			32.41 ± 4.40			44.70 ± 5.88		
Nationality **									
Saudi	47.96 ± 5.82	<0.001	0.9	32.58 ± 6.64	0.027 *	0.36	44.09 ± 5.49	0.003	0.34
Non-Saudi	43.09 ± 4.89			28.86 ± 3.73			42.32 ± 4.81		
PHC work experience *									
< 5 years	47.58 ± 5.80	0.014		32.26 ± 6.40	<0.001		43.80 ± 5.39	0.204	
5–10 years	44.48 ± 6.32		0.78	30.21 ± 5.05		0.64	42.74 ± 5.32		0.23
> 10 years	43.22 ± 4.26			28.68 ± 4.15			42.69 ± 3.62		
Current position *									
Resident	45.57 ± 5.67	0.104		30.66 ± 5.55	0.131		43.01 ± 4.62	0.736	
Specialist	44.40 ± 5.75		0.41	29.82 ± 5.47		0.34	43.25 ± 5.09		0.11
Consultant	46.91 ± 6.87			32.30 ± 6.06			43.73 ± 6.15		

* One-way ANOVA test applied. ** Independent *t*-test applied.

**Table 4:** Correlation between the knowledge, attitude, and practice towards DR.

Practice towards DR		
	Correlation Coefficient (*r*) *	*p*-Value
Knowledge	0.739	<0.001
Attitude	0.479	0.007

* Pearson correlation coefficient.

**Table 5:** Association between the knowledge, attitude, and practice towards DR assessed by a multilinear regression (adjusted for other covariables).

	Practice towards DR *	
	Regression Coefficient (95% Confidence Interval)	*p*-Value
Knowledge	0.658 (0.571–0.735)	0.008 **
Attitude	0.539 (0.380–0.681)	0.038 **

* Adjusted covariables: gender, age group, current position, qualification, nationality, PHC work experience duration. ** Statistically significant value.

## Data Availability

The data presented in this study are available on request from the corresponding author.
